# Computational Analysis of Heat Capacity Effects in Protein–Ligand Binding

**DOI:** 10.1021/acs.jctc.4c00525

**Published:** 2024-06-13

**Authors:** Lucien Koenekoop, Johan Åqvist

**Affiliations:** Department of Cell & Molecular Biology, Uppsala University, Biomedical Center, SE-751 24 Uppsala, Sweden

## Abstract

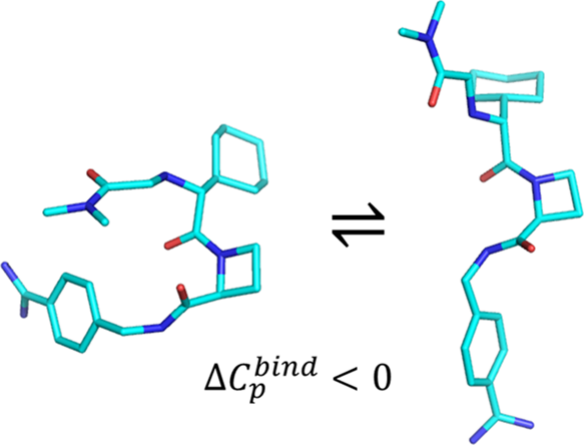

Heat capacity effects in protein–ligand binding as measured by calorimetric experiments have recently attracted considerable attention, particularly in the field of enzyme inhibitor design. A significant negative heat capacity change upon ligand binding implies a marked temperature dependence of the binding enthalpy, which is of high relevance for attempts to optimize protein–ligand interactions. In this work, we address the question of how well such heat capacity changes can be predicted by computer simulations. We examine a series of human thrombin inhibitors that all bind with Δ*C*_p_ values of about −0.4 kcal/mol/K and calculate heat capacity changes from plain molecular dynamics simulations of the bound and free states of the enzyme and ligand. The results show that accurate Δ*C*_p_ estimates within a few tenths of a kcal/mol/K of the experimental values can be obtained with this approach. This allows us to address the structural and energetic origin of the negative heat capacity changes for the thrombin inhibitors, and it is found that conformational equilibria of the free ligands in solution make a major contribution to the observed effect.

## Introduction

The observation of temperature-dependent thermodynamic parameters in protein–ligand binding studies and drug discovery has received much attention in recent years.^1,2^ Particularly, the temperature dependence of the binding enthalpy from isothermal titration calorimetry (ITC) experiments is used to quantify the heat capacity change upon ligand binding (Δ*C*_p_), which is typically negative by a few tenths of a kcal/mol/K in cases where the enthalpy shows any significant temperature dependence.^3−6^ Such heat capacity effects have often been ascribed to solvation/desolvation of nonpolar and polar groups and hydrophobic interactions.^4,6−9^ Another hypothesis is that conformational restriction or redistribution of vibrational modes in the protein–ligand complex is what causes a drop in the heat capacity.^6,9−11^ In addition, Eftink and co-workers elegantly showed how conformational and other equilibria involving the free protein and/or ligand inevitably will give rise to heat capacity changes associated with binding^12^ (see also ref (2)). This will be discussed further below. Both the interpretation in terms of solvation/desolvation and loss of conformational degrees of freedom when the ligand binds can be seen as analogies with protein folding, which is also accompanied by a considerable drop in *C*_p_.^8^ The issue of temperature-dependent binding enthalpies (Δ*H*) is also of particular concern in drug discovery where the optimization of Δ*H* is often considered to be a viable strategy for attaining high affinity and selectivity.^1^

The question we want to address herein is whether the heat capacity change upon ligand binding can be reliably predicted by molecular dynamics (MD) simulations and, if so, what is its origin? Ideally, one would like to be able to calculate the absolute binding free energy for a given ligand at a series of different temperatures and fit the results to the expression
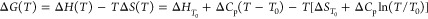
1where *T*_0_ is an arbitrary reference temperature (usually taken as 25 °C). This equation is approximate in the sense that Δ*C*_p_ is assumed to be constant over the relevant temperature range. Fitting free energies to eq 1 would then, in addition to Δ*C*_p_, yield the desired values of Δ*H* and Δ*S* at any given temperature. Unfortunately, such an approach is not really viable due to the relatively large errors associated with absolute binding free-energy calculations from MD simulations.^13,14^ An ambitious attempt to calculate the temperature dependence of absolute ligand binding free energies has, however, been reported by Deng et al.,^15^ who could thereby distinguish the thermodynamic signatures of polar and nonpolar binding. Protein–ligand binding free-energy calculations are still mostly used for obtaining relative binding free energies, and if such simulations are carried out at different temperatures, one could thus, in principle, extract (relative) values of ΔΔ*H*, ΔΔ*S*, and ΔΔ*C*_p_.^16−18^ However, even calculations of relative binding free energies (ΔΔ*G*) are often plagued by error bars that are too large to obtain accurate relative van’t Hoff plots and, particularly, to observe a small curvature in these that would be associated with a ΔΔ*C*_p_ ≠ 0.

Interestingly, the situation is rather different for calculations of Arrhenius plots for chemical reactions in enzymes and solution (Δ*G*^⧧^ vs *T*), where we have shown that the temperature dependence can be very accurately captured by MD simulations using the empirical valence bond (EVB) method.^19−23^ The underlying reason for this difference between reaction and binding free-energy calculations is that the perturbations involved in the former are much smaller than in the latter. That is, in reaction simulations, atoms do not vanish or appear as in alchemical free-energy calculations but only the charge distribution and bonding changes between a fixed set of atoms. In relative binding free-energy calculations, on the other hand, groups of atoms usually differ between ligands and are treated as appearing and disappearing along the free-energy perturbation path. The same goes for calculations of the effects of protein mutations on ligand binding or protein stability where, e.g., mutation of an alanine residue to tryptophan or of a neutral residue to a charged one usually causes the error bars to increase beyond what would be needed for accurate van’t Hoff plots.

**Figure 1:**
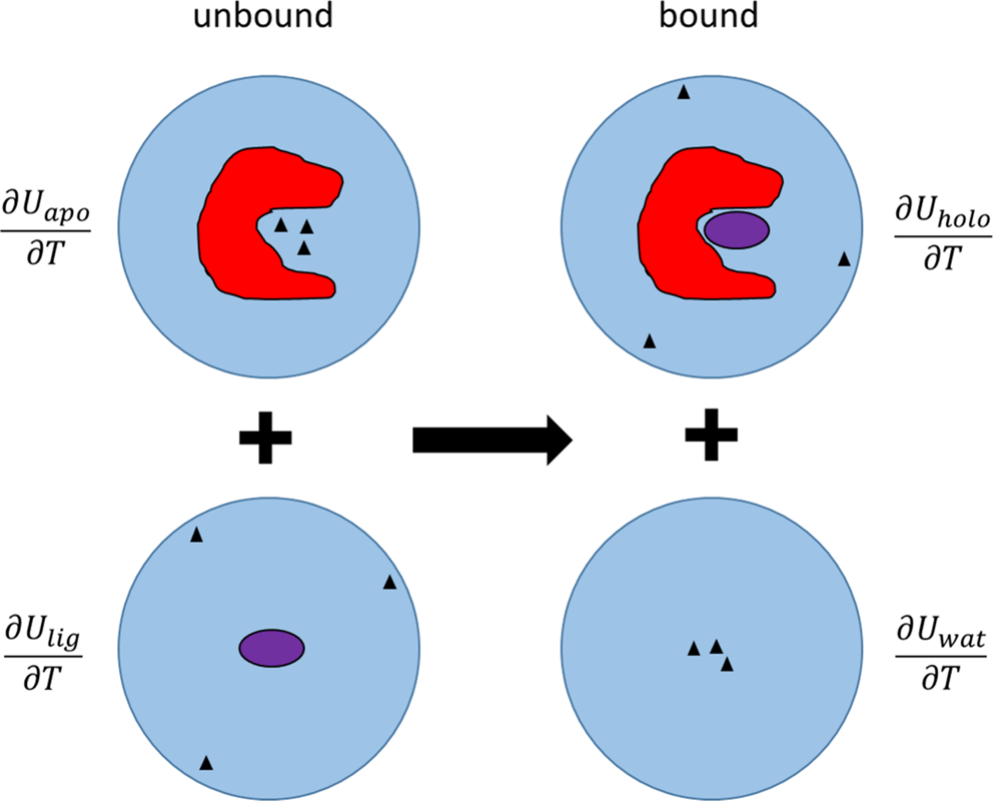
Thermodynamic process describing the calculation of binding heat capacities from four different MD simulations: apoenzyme, holoenzyme, free ligand in water, and pure water. For each separate system, the derivative of the total energy is calculated as a function of temperature. Black triangles schematically denote solvent molecules that are displaced by the ligand. The total number of solvent molecules is kept constant both in the upper and lower processes so that the overall process does not involve any change in the number of degrees of freedom.

The possible heat capacity change associated with ligand binding can, however, in principle, be obtained, without any free-energy calculations, from the much simpler temperature derivatives of the enthalpy in the bound and unbound states
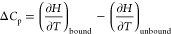
2where the enthalpy can be replaced by the average total energy ⟨*E*_tot_⟩ or potential energy ⟨*U*_tot_⟩. As pointed out earlier,^24^ it is then essential that the two systems that are compared (bound and unbound) have exactly the same number of degrees of freedom since each of them adds a contribution to the heat capacity of the system. We thus devised the scheme shown in Figure 1, where two separate processes are combined.^24^ The first represents going from the apoprotein to the protein–ligand complex in solution, and the second represents removing the ligand from the state where it is free in solution. Ideally, the number of water molecules for the two systems in the upper half of the diagram should be equal and likewise for the two systems without any protein in the lower half. This ensures that the number of degrees of freedom is equal in the representation of the binding process. However, if the number of waters is not exactly identical in the apo- and holoprotein systems, a correction for this can be added since their individual contribution to *C*_p_ can easily be calculated for bulk water.^24^

The procedure described above (Figure 1) was earlier examined for the binding of a 6-nitrobenzisoxazole substrate to a Kemp eliminase designer enzyme and showed that Δ*C*_p_ = 0 in that case.^24^ This appears to be the normal situation for most proteins, with no appreciable heat capacity change upon ligand binding and the corresponding van’t Hoff plots would thus be perfectly linear. In order to further examine the scope of this computational strategy for heat capacity calculations, we looked for experimentally determined cases with significant nonzero values of Δ*C*_p_. To this end, we chose a set of human thrombin inhibitors where Δ*C*_p_ has been found to be about −0.4 kcal/mol/K.^5^ While such a numerical value may seem small, it is sufficient to cause a substantial temperature dependence of the binding enthalpy and entropy.^5,6^ Hence, this magnitude of Δ*C*_p_ implies that Δ*H* will change by about 10 kcal/mol over a temperature range of 25 °C, which is rather remarkable. For the thrombin inhibitors examined herein, our results show that the absolute value of Δ*C*_p_ can be reliably reproduced to within about 0.2 kcal/mol/K, with very reasonable computer simulation times. This allows us to address the origin of the heat capacity effect on an atomistic scale, and our conclusion is that conformational equilibria between compact and extended ligand structures, when it is free in solution, seem to play a major role in determining Δ*C*_p_ for these relatively large inhibitors.

## Results

### Heat Capacity Calculations

**Figure 2:**
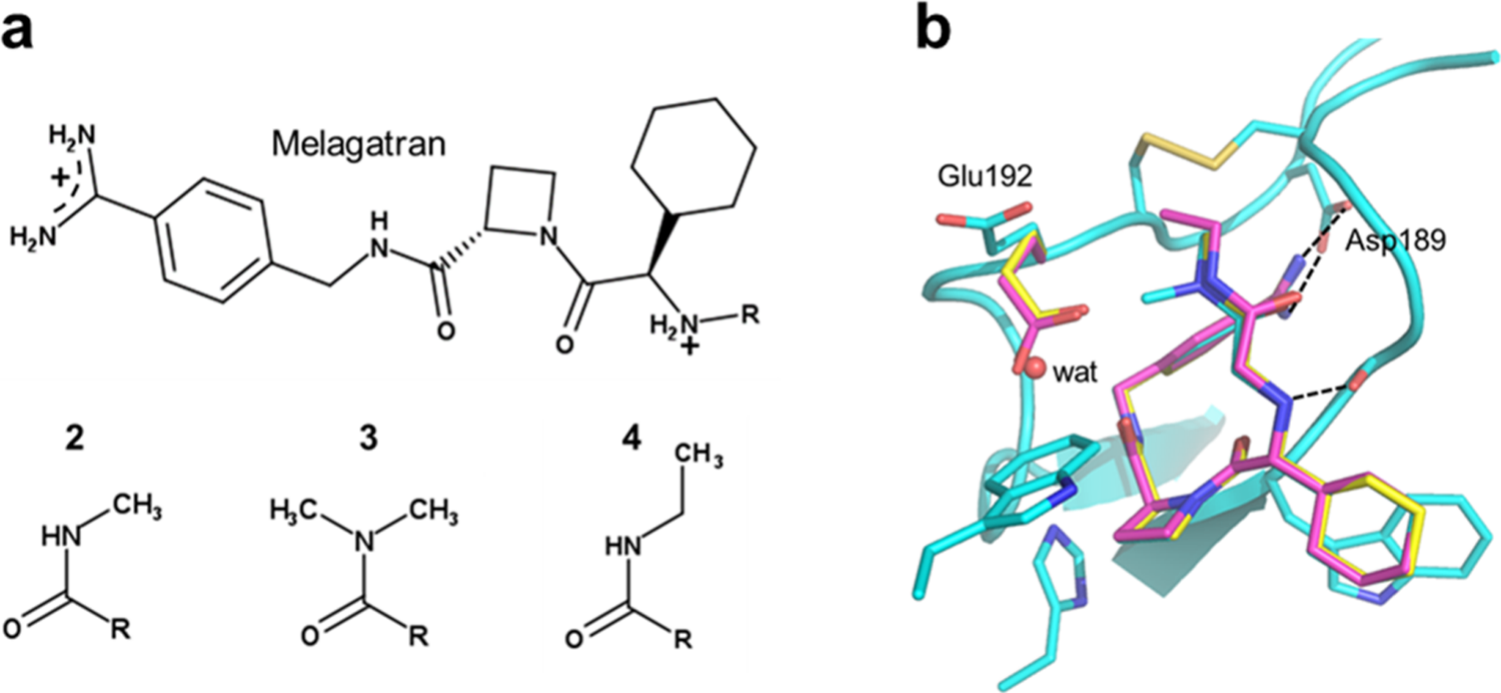
(a) The three Melagatran derivatives (**2**, **3**, **4**) considered in this work. (b) Crystallographic structures of the three inhibitors bound to the active site of human thrombin (yellow = **2**, cyan = **3**, pink = **4**).^5^ The only noticeable difference between the three complexes is that Glu192 is pushed away by the larger substituent of **3**, which allows the entry of one additional water molecule (red sphere).

The three thrombin inhibitors considered here (Figure 2a) are all derivatives of the anticoagulant drug Melagatran and bind to the enzyme with nM affinity (Δ*G*_bind_ ∼ −12 kcal/mol).^5^ They bind to the active site in a well-characterized binding mode where the inhibitor benzamidine group forms an ion pair with Asp189 deep in the S1 pocket of the active site, thereby blocking access to the catalytic triad.^5,25^ The crystal structures of the three inhibitor complexes are very similar, and the only real difference between them is a rotation of the Glu192 side chain caused by the branched substituent of compound **3**, accompanied by the entry of one extra water molecule near it (Figure 2b).^5^ The three inhibitors also have very similar experimentally determined values of Δ*C*_p_: −0.41, −0.42, and −0.39 kcal/mol/K, respectively, for compounds **2**, **3**, and **4**.^5^ Nevertheless, this is a good and relevant test case since we are mainly interested in determining the reliability of absolute Δ*C*_p_ calculations. The crystal structures for each of the three ligands were used as starting points for the MD simulations, and the apoprotein was modeled by removing compound **3** from its complex with thrombin (pdb code 4BAM).^5^ The crystal structures all contain the short hirugen peptide (10 residues in the crystallographic models) bound to exosite I of thrombin. This peptide is often used in crystallization and contributes to neutralization of the otherwise positively charged protein. In our simulations, we considered two different models, one with hirugen present and one with it absent. In the latter case, the apoprotein was instead neutralized by the inclusion of Cl^–^ counterions and this system apparently corresponds more closely to the experimental conditions of the ITC measurements.^5^ Data was collected for each system during a total of 150–250 ns of MD simulation at five different temperatures using a spherical droplet that covers the entire protein.

**Figure 3:**
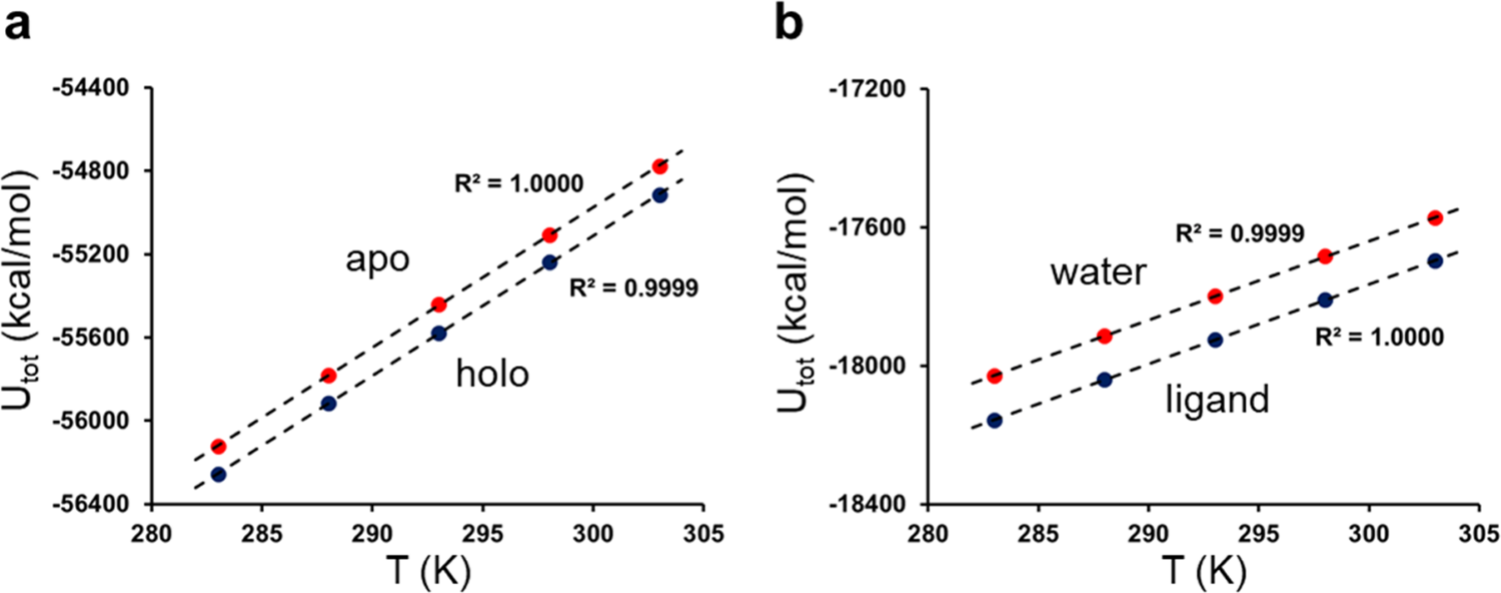
Calculated average total potential energies for ligand **3** as a function of temperature for (a) the apo- and holoenzymes (without hirugen bound) and (b) for the ligand in water and for pure water. The corresponding plots for ligands **2** and **4** are virtually indistinguishable.

The MD simulations yield essentially perfectly linear relationships between the average total potential energy ⟨*U*_tot_⟩ and temperature, both for the apoenzyme and for its complexes with the three inhibitors (Figure 3). Hence, the *R*^2^ values for these plots are all equal to 1, to within the third decimal, and the quantity ∂*U*/∂*T* is thus very well-defined. Moreover, this indicates that the heat capacity for each system is relatively constant over the temperature interval and does not change appreciably. Taking the relevant difference between these derivatives (Figure 1)
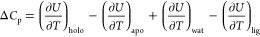
3thus gives our estimate of the heat capacity change upon binding the different ligands.^24^ Remarkably, all three compounds show negative values of Δ*C*_p_ of the correct magnitude^5^ and the values range from −0.56 kcal/mol/K for compound **2** to −0.19 kcal/mol/K for compound **4** (Table 1). It can be seen that the errors with respect to the experimental values range from 0.15 to 0.20 kcal/mol/K for the calculations without hirugen. Also, with hirugen bound to the enzyme, all Δ*C*_p_ values are similarly negative.

**Table 1:** Calculated Heat Capacities (kcal/mol/K) for the Different Systems and the Total Binding Δ*C*_p_^a^

inhibitor					Δ*C*_p_^calc^	Δ*C*_p_^expt^
2 (−hir)	66.962	67.307	23.083	22.871	–0.56 ± 0.01	–0.41 ± 0.03
2 (+hir)	66.699	66.857	23.083	22.871	–0.37 ± 0.01	–0.41 ± 0.03
3 (−hir)	67.191	67.307	23.072	22.871	–0.32 ± 0.01	–0.42 ± 0.01
3 (+hir)	66.649	66.857	23.072	22.871	–0.41 ± 0.01	–0.42 ± 0.01
4 (−hir)	67.310	67.307	23.063	22.871	–0.19 ± 0.01	–0.39 ± 0.03
4 (+hir)	66.829	66.857	23.063	22.871	–0.22 ± 0.01	–0.39 ± 0.03

a Error bars (asymptotic standard error) for the derivatives from linear regression are ±0.003 to ±0.009 kcal/mol/K from the protein simulations and ±0.002 to ±0.003 kcal/mol/K for the simulations in water. ±hir denotes whether hirugen is bound or not. The ∂*U*/∂*T* value obtained for pure water yields a *C*_p_ = 0.0179 kcal/mol/K per molecule when corrected with the 3*R* kinetic energy term, in good agreement with the experimental value (0.018 kcal/mol/K).^24^

### Free-Energy Perturbation Calculations

Since the three inhibitors have slightly different binding free energies and enthalpies, as measured by stopped-flow and SPR experiments,^5^ it is also interesting to examine the performance of FEP calculations in this case. To this end, we calculated the binding free-energy difference (ΔΔ*G*_bind_) between compounds **2** and **4**, which only differ by one methyl group, as a function of temperature (Supporting Information). These results show that even if the predicted values of ΔΔ*G*_bind_ around −0.3 kcal/mol agree fairly well with the experimentally measured quantity (ΔΔ*G*_bind_ = −0.39 kcal/mol at 25 °C),^5^ the calculated temperature dependence of ΔΔ*G*_bind_ is not accurate enough for reliable estimation of Δ*C*_p_ differences between ligands (Figure S1).

### Do the Negative Δ*C*_p_ Values Derive from Differences in Protein Structure and Mobility?

**Figure 4:**
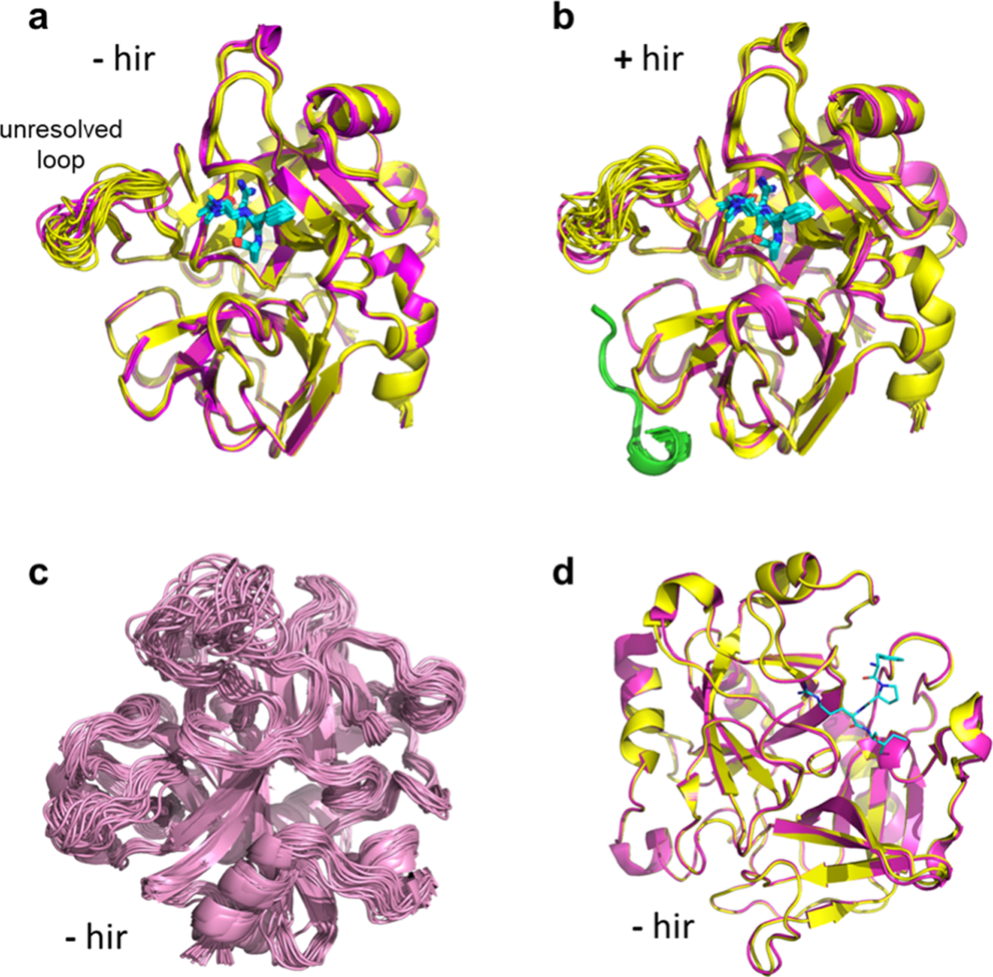
Average MD structures of the apoenzyme (pink) and the complexes (yellow) with the three ligands (cyan), overlaid at all five temperatures without (a) and with (b) hirugen bound (green). The crystallographically unresolved loop region is indicated. (c) Illustration of the conformational sampling of the apoenzyme by overlaying 30 snapshots at 298 and 303 K (interspaced by 1 ns). (d) Superimposed crystallographic apo (pink) and holo (yellow–inhibitor in cyan) structures of thrombin from ref (28)

Since our calculations of absolute Δ*C*_p_ values turn out to be remarkably accurate compared to the experimental results, the microscopic origin of these negative binding heat capacities is very interesting to try to understand. The first obvious quantities to look at would be the average structures of the holo and apo forms of the enzyme and their corresponding atomic fluctuations. Figure 4a,b shows overlays of the average apo and holo structures for the three ligands at each of the five temperatures, where it can be seen that all of the average structures are very similar. There is also no major difference if hirugen is absent (Figure 4a) or present (Figure 4b). In fact, the r.m.s. Cα coordinate deviation between all of the different average structures and the crystal structure of compound **2** is in all cases ≤0.7 Å for the complexes and ≤0.8 Å for apo structures. This clearly demonstrates that the average enzyme structures with different ligands and at different temperatures are very stable, although considerable flexibility is observed in the MD simulations as expected (Figure 4c).^26^ The only part of the structure that shows some degree of variability in the average structures is the 7-residue loop region between Thr147 and Gly150 (thrombin numbering by Bode et al. is used throughout),^27^ but there is no systematic conformational trend between holo and apo structures here. This loop region is also missing in the crystallographic models,^5^ presumably due to its high flexibility (see below). It should further be pointed out that the experimental structure of the apoenzyme,^28^ which also lacks the loop region, has a Cα coordinate deviation of only 0.4 Å (275 heavy and light chain Cα’s) with respect to the crystallographic holo structures used here.^5^ Hence, we can conclude that structures of the ligand-bound and ligand-free forms of thrombin are extraordinarily similar both in our MD simulations and from crystallographic experiments (Figure 4d).

**Figure 5:**
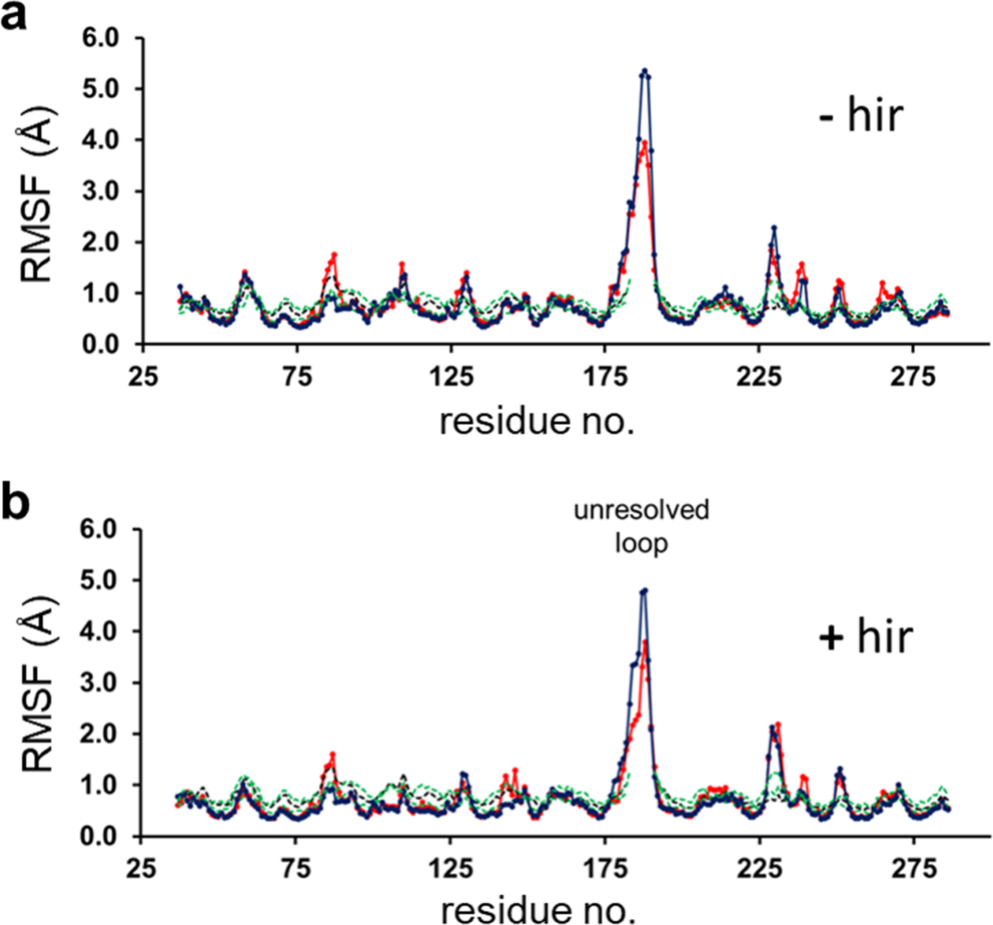
Calculated average backbone r.m.s. positional fluctuations per residue along the amino acid sequence at 303 K for the apoenzyme (red curve) and the complex with ligand **3** (blue curve), (a) without and (b) with hirugen bound. Also shown are the corresponding RMSF curves calculated from the crystallographic *B*-factors of the inhibitor complexes 4BAM(5) and 3U8R(28) (dashed green) and the apo structure 3U69(28) (dashed black). Note that the contiguous sequence numbering here is that of PDB entry 4BAM.^5^

The r.m.s. atomic coordinate fluctuations around the average structures (RMSF) calculated from the MD simulations also turn out to be extremely similar between the different systems. That is, at each temperature, the RMSFs for the different ligand complexes show no significant differences when compared to the corresponding apo structure (Figure 5). It can be noted that the above-mentioned loop region involving residues 141–155 (177–190 in Figure 5) is in all cases the most mobile part of the structure and it thus makes sense that this region is not well resolved in the electron density maps. There is a small increase in the mobility of the loop region around residue 86 (Trp60D in the Bode et al. numbering)^27^ for the apo structures (both MD and X-ray), which presumably reflects the fact that this tryptophan side chain makes contact with the inhibitors. The average MD structures, however, do not show any significant differences in this region. Likewise, the MD structures (both apo and holo) show a slightly increased mobility around residue 229 (Asp186A), which appears affected by crystal contacts in the experimental structures. In general, however, the agreement between calculated and experimental RMSFs is very good. We can thus conclude that differences in protein flexibility between the different complexes and the unliganded enzyme do not either seem to explain the origin of the negative binding heat capacities. In fact, Winquist et al.^5^ noted that since the Δ*C*_p_ values were statistically indistinguishable between the different ligand complexes, the negative value(s) is not likely to derive from the P3 substituent of the ligands, which is the only part that differs. Our analysis, so far, indicates that the negative Δ*C*_p_ does not either derive from the S1 and S2 pockets of the enzyme since the structures and RMSFs compared to the apoenzyme do not really show any differences in the simulations.

### The Conformational Equilibrium Model for Heat Capacity Changes

As was pointed out already 40 years ago by Eftink and co-workers,^12^ heat capacity effects on protein–ligand binding can also be explained in terms of conformational equilibria, as opposed to an “intrinsic” heat capacity difference between the bound and dissociated systems. Such equilibria can involve either the unliganded protein, or the ligand itself, and may also pertain to protonation states, protein or ligand aggregation, etc.^12^ We have further proven that the existence of such a conformational equilibrium is responsible for the apparent negative heat capacity associated with *k*_cat_ (Δ*C*_P_^⧧^) for a cold-adapted α-amylase.^20,29^ In that case, a catalytic rate optimum occurring well below the enzyme melting temperature arises due to the increased population of an inactive enzyme–substrate state at higher temperatures.^20,29^ So, could it be possible that the observed negative binding heat capacities for the six Melagatran analogues examined in ref (5) also reflect some type of conformational equilibrium, rather than an intrinsic heat capacity difference caused by rigidification of the protein structure by the ligands (for which we see no evidence here)? What speaks in favor of a conformational equilibrium involving either the apoenzyme or the ligands themselves is that the measured Δ*C*_p_ values for all six inhibitors are about −0.4 kcal/mol/K.^5^

It is therefore useful to examine the predictions of an equilibrium model obtained by fitting the experimentally measured binding enthalpies from calorimetry.^5^ That is, we can use the expression for the apparent binding enthalpy^12,20^
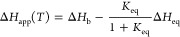
4resulting from the scheme , where *E* + *L* denotes the binding competent state of the free enzyme (*E*) and ligand (*L*), while (*E* + *L*)′ is an inactive state that differs either in the conformation of the enzyme or the ligand. Hence, this scheme represents what Eftink et al. called “mandatory coupling”,^12^ meaning that only the *E* + *L* state can form the bimolecular complex. Here, *K*_b_ is thus the true binding constant for *E* + *L*, while *K*_app_ = *K*_b_/(1 + *K*_eq_) is the apparent binding constant that is reduced by populating the inactive state.

**Figure 6:**
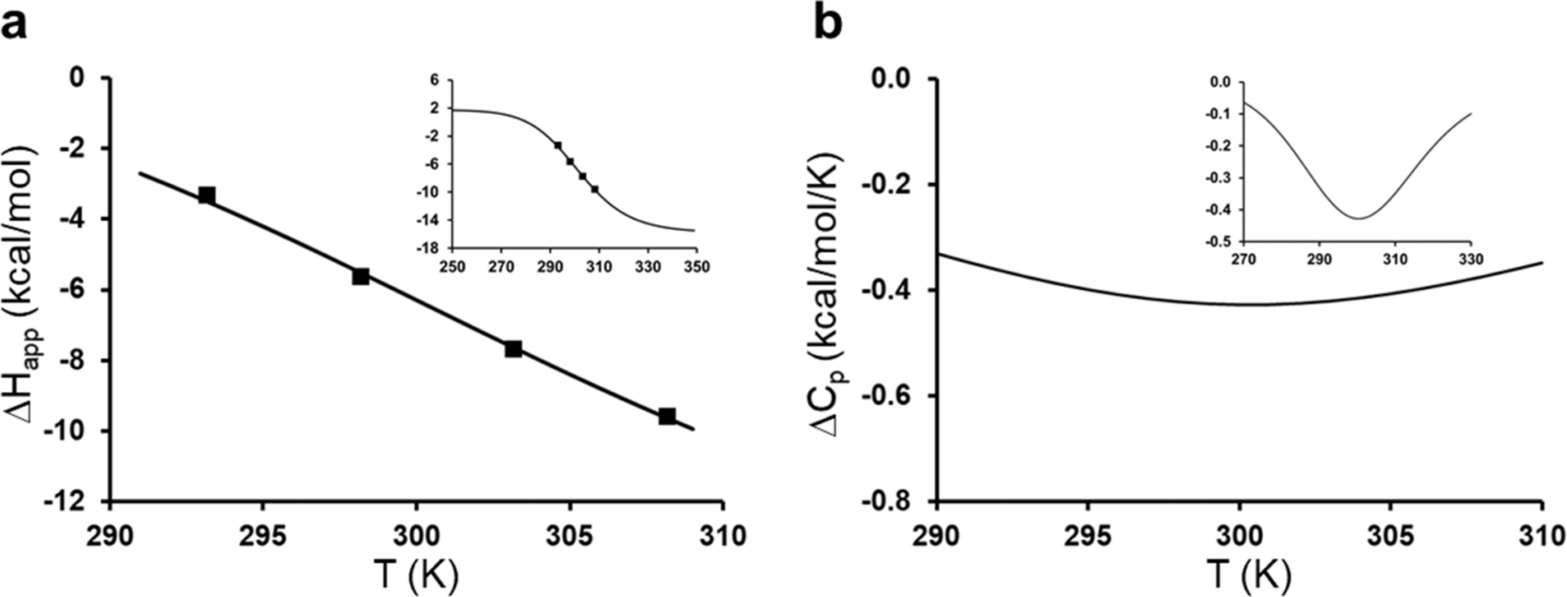
(a) Temperature dependence of the apparent binding enthalpy for inhibitor **3** predicted by the equilibrium model (eq 4), using the average fitted values of Δ*H*_eq_ and Δ*S*_eq_ for all six ligands in ref (5). Experimental ITC data points for ligand **3** are shown as black squares. (b) Temperature dependence of the apparent binding heat capacity obtained from the average values of Δ*H*_eq_ and Δ*S*_eq_. The insets show the behavior of Δ*H*_app_ and Δ*C*_p_ over a larger temperature interval.

It can be seen from Table 2 that the six ligands yield very similar values of the thermodynamic parameters for the hypothetical equilibrium, which is, of course, logical in view of their similar apparent Δ*C*_p_ values. The average values over all six ligands are Δ*H*_eq_ = 17.57 ± 0.13 kcal/mol and Δ*S*_eq_ = 0.0582 ± 0.0005 kcal/mol/K, where the errors denote the s.e.m. Using these average values for the equilibrium yields an accurate representation of how the apparent binding enthalpies for the different ligands vary with temperature (Figure 6a). Moreover, as shown in the inset of Figure 6a, the asymptotic behavior of Δ*H*_app_, with limiting values at low and high temperatures, is clearly more reasonable than the monotonic behavior predicted by eq 1 with a constant Δ*C*_p_. The equilibrium model instead predicts a temperature-dependent Δ*C*_p_ that is given by
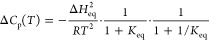
5where the two rightmost factors denote the probability of being in the active and inactive states, respectively. The smaller the magnitude of Δ*H*_eq_ and Δ*S*_eq_ is, the flatter the Δ*C*_p_ (*T*) curve will be and the magnitude of its characteristic dip will also become smaller. This is illustrated for our case in Figure 6b, where it can be seen that Δ*C*_p_ varies only marginally over the temperature interval 290–310 K, which roughly corresponds to the experimental ITC measurement interval.^5^ Hence, this can essentially be interpreted as a constant Δ*C*_p_ over the given temperature interval.

**Table 2:** Parameters Obtained from Fitting the Equilibrium Model of Eq 4 to the Data of Ref (5) (Units in kcal/mol and kcal/mol/K)

ligand^a^	Δ*H*_eq_	Δ*S*_eq_	Δ*H*_b_	Δ*S*_b_	Δ*H*_app_^298^	Δ*H*_exp_^298^	Δ*G*_eq_^298^	Δ*G*_b_^298^	Δ*G*_app_^298^
**0**	17.89	0.0590	0.07	0.0422	–6.64	–6.65	0.30	–12.50	–12.22
**1**	17.49	0.0579	1.86	0.0467	–5.24	–5.03	0.23	–12.06	–11.75
**2**	17.63	0.0583	2.21	0.0486	–4.81	–4.55	0.24	–12.29	–11.99
**3**	17.86	0.0601	3.69	0.0559	–5.57	–5.61	–0.04	–12.97	–12.54
**4**	17.02	0.0565	1.70	0.0491	–5.50	–5.21	0.18	–12.93	–12.61
**5**	17.50	0.0575	1.50	0.0477	–4.76	–4.55	0.35	–12.74	–12.47
⟨**0–5**⟩	17.57	0.0582					0.21		
alternative solution:^b^ Δ*H*_eq_^′^ = −Δ*H*_eq_, Δ*H*_b_^′^ = Δ*H*_b_ – Δ*H*_eq_									
**5**	–17.50	–0.0575	–16.00	–0.0098	–4.76	–4.55	–0.35	–13.08	–12.47
⟨**0–5**⟩	–17.57	–0.0582					–0.21		

a The data for all six ligands of ref (5) is considered here.

b As eq 4 always has two solutions in terms of Δ*H*_b_, Δ*H*_eq_, and Δ*S*_eq_, this alternative solution is given as an illustration for ligand **5**.

As noted earlier, eq 4 always has two solutions (Table 2) that differ in the signs of Δ*H*_eq_ and Δ*S*_eq_, with a simple relationship for the corresponding values of Δ*H*_b_ and Δ*S*_b_ (Δ*H*_b_^′^ = Δ*H*_b_ – Δ*H*_eq_ and Δ*S*_b_^′^ = Δ*S*_b_ – Δ*S*_eq_).^30^ The first solution has positive values of Δ*H*_eq_ and Δ*S*_eq_, implying that the inactive state is uphill at low temperature and downhill above the transition point at ∼29 °C, given by the current model. In this case, the true binding free energy Δ*G*_b_ is strongly entropy-driven with a small positive value of Δ*H*_b_. The alternative solution has the quantities reversed so that the equilibrium instead now favors the inactive state at low temperature, while Δ*G*_b_ becomes enthalpy-driven with an entropy penalty of 1–5 kcal/mol at 25 °C for the different inhibitors. It is, of course, difficult to *a priori* say anything about which type of equilibrium would be most reasonable. On the other hand, the predictions of the above models can give some clues regarding what to look for in the MD simulations.

### The Conformational Equilibrium Involves the Free Ligands in Solution

**Figure 7:**
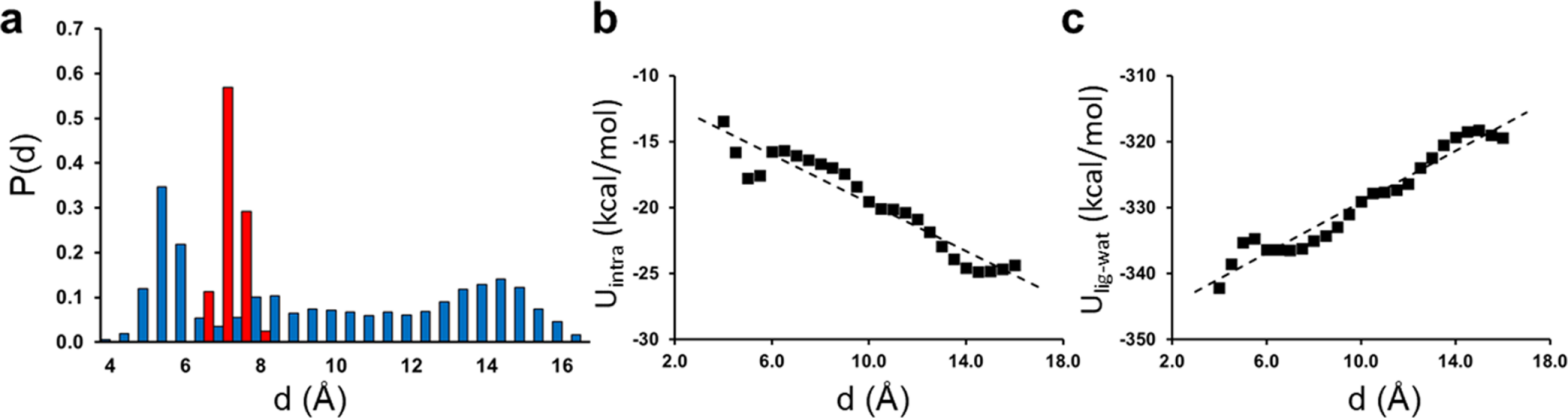
(a) Probability distribution for the end-to-end distance of ligand **3** in water at 25 °C (blue bars) compared to the corresponding distribution in its complex with thrombin (red bars). The average intramolecular (b) and intermolecular (ligand–water) interaction energy (c) for inhibitor **3** in water at 25 °C as a function of the end-to-end distance.

Since we do not see any signs of significant conformational changes between the holo and apo forms of the enzyme (Figure 4), we should also consider the possibility that it could be the free ligands in solution that display some type of structural transition. Indeed, it turns out that the free ligands sample a distribution of conformations that can be best characterized in terms of their end-to-end distance. There is typically a distinct peak around 5.5 Å, corresponding to a compact structure similar to that found in the complexes with thrombin, and a broader peak at *d* > 11 Å where the structure is extended (Figure 7a). At low temperatures, the population of the compact conformer increases, while at high temperature, it decreases and the peak associated with the extended conformation broadens and that type of structures becomes dominating. Judging from the crystal structures of the complexes, as well as from our average MD structures (Figure 4a,b), it seems clear that the compact structures can readily bind to the active site, while the extended conformation cannot. Hence, the extended ligand conformations can tentatively be assigned to the inactive state in the above equilibrium model.

The two types of ligand conformations are also associated with different energetic behaviors. That is, as the end-to-end distance increases, the nonbonded intramolecular ligand interactions become more favorable, while the intermolecular interactions with the solvent become less favorable. This effect is completely dominated by electrostatics and can be understood in terms of the interaction between two positively charged ligand groups and their solvation by water (Figure 7b,c). As the distance between them increases, their mutual repulsion decreases, and the intramolecular interaction energy becomes more favorable. In contrast, the attractive interactions with water diminish as the charges become less “concentrated” and, hence, the charge stabilization by water weakens when the structure becomes more extended (compare the solvation free energy of two Na^+^ ions to that of one Ca^2+^ ion). As can be seen in Figure 7b,c, the latter ligand–water interactions dominate the overall energetics, suggesting that the extended conformation is uphill in energy. The water–water interactions, of course, add a contribution to this picture and become more favorable when the charges become separated, but the overall effect is that the extended conformations are uphill in enthalpy compared to compact ones.

Based on the analysis above, it thus appears that the solution of the equilibrium model with positive values of Δ*H*_eq_ and Δ*S*_eq_ is the most reasonable one. This would thus imply that the true binding free energies of the compact structures are strongly driven by a favorable binding entropy (Δ*S*_b_ > 0), whereas the corresponding enthalpy (Δ*H*_b_) is generally positive by a few kcal/mol. This situation can be interpreted such that the interactions with the two charged ligand groups in the complex are not sufficiently strong to compensate for their lost interactions with water. This appears especially true for the secondary nitrogen of the P3 moiety that only has one hydrogen bond from the protein, with the backbone carbonyl of Gly255, while the benzamidine group forms an ion pair with Asp189 (Figure 2b). In addition, the burial of hydrophobic moieties may also contribute to a positive binding entropy.

## Discussion

In this work, we have shown that a straightforward method for calculating heat capacity changes in protein–ligand binding performs remarkably well for a set of experimentally characterized thrombin inhibitors with relatively large heat capacity effects. These ligands all bind with an apparent Δ*C*_p_ of about −0.4 kcal/mol, which is enough to cause a major change in the apparent binding enthalpy of about 10 kcal/mol over a temperature range of 25 °C, as observed in the ITC experiments.^5^ Our calculations yield negative binding heat capacities for all three ligands that are within 0.2 kcal/mol/K of the experimentally determined Δ*C*_p_ values. As a comparison, we also showed that computational van’t Hoff plots of relative binding free energies versus temperature are not likely to be accurate enough to determine heat capacity changes, although they give very reasonable free energies. Analysis of the average MD structures of the ligand complexes and the free enzyme at different temperatures do not provide any clear clues regarding the origin of the negative Δ*C*_p_ values. The same goes for the calculated atomic positional fluctuations, which are also found to be extraordinarily similar between the apo- and holoenzymes.

To shed further light on the observed heat capacity effects, we examined a conformational equilibrium model, where the equilibrium could pertain either to the free enzyme or to the free ligand in solution.^2,12^ This equilibrium model makes excellent predictions for both the apparent binding enthalpy and heat capacity, and our structural and energetic analysis clearly suggests that the conformational behavior of free ligand in solution plays a major role here. That is, the inhibitors sample a wide range of conformations in solution that span between very compact structures and those that are more extended. The reduced heat capacity accompanying ligand binding would thus reflect the elimination of this equilibrium when the ligand is taken out of water and into the protein binding site. The magnitude of this effect can also be seen in Table 1 to be about −0.2 kcal/mol/K in terms of (∂*U*/∂*T*)_wat_ – (∂*U*/∂*T*)_lig_. This contribution does, however, not seem to account for the full value of Δ*C*_p_, and, in this respect, it may be useful to also consider the effect of displacing water molecules from the protein binding site. What is interesting here is that water molecules hydrating a protein may have a significantly higher heat capacity than bulk water,^12,31^ so that the release of such waters may also decrease *C*_p_ of the bound state. With regard to the flexible surface loop adjacent to the ligand binding site, it cannot be excluded that its mobility also makes a contribution to the reduced *C*_p_ of the holoenzymes, although we have not found any systematic trend here.

It is, of course, possible that binding heat capacity changes for other protein–ligand systems may have a different origin than our thrombin case, in particular, for ligands that can rigidify otherwise highly flexible proteins. Nevertheless, it is certainly an interesting question to what extent observed nonzero Δ*C*_p_ values, in general, actually originate from different types of equilibria that are suppressed upon binding. The results presented herein, as well as earlier work that analyzed the available experimental data,^2,12^ do suggest that different types of equilibria can play a major role in causing negative binding heat capacities, possibly being more important than intrinsic *C*_p_ differences between the bound and free states of the protein. If so, the analogy to the negative heat capacity change in protein folding, which is more akin to a phase transition, could be somewhat misleading. At any rate, it is clear that computer simulations can provide a straightforward route for answering this interesting question.

## Methods

### System Preparation

The structural models for thrombin were based on refined crystal structures in complex with the Melagatran analogues found in the PDB entries 4BAN, 4BAM, and 4BAQ, denoted compounds **2**, **3**, and **4**, respectively.^5^ These analogues all have the P3 carboxylate group substituted with an amide that is terminated with dimethyl-, methyl-, and ethylamine groups, respectively. Structure preparation was done with Schrödinger’s Maestro (Schrödinger Suite Release 2021–1, v12.7.161)^32^ to check for asparagine and glutamine flips and histidine protonation states (at pH 7.0), along with p*K*_a_ predictions by PropKa (v3.1).^33^ Apart from the sodium ion nestled between the 220-loop and the 186-loop,^34^ other heterogroups were removed from the structure, as well as the terminal residues with missing side-chain atoms Asp14L (C-terminal of light chain) and Phe245 (original thrombin numbering by Bode et al.).^27^ The seven missing loop residues between Thr147 and Gly150 were filled in with Prime.^32^ Simulations were performed under spherical boundary conditions where the center of mass of the complex was chosen as the center of the system. The simulation systems included the three thrombin–ligand complexes, the apoenzyme, the three ligands free in water, and a plain water sphere. In addition, the simulations of the apoenzyme and the three complexes were carried out both with and without the 10-residue hirugen peptide that is present in the experimental structures.^5^ In the former case, the tyrosine residue was not sulfatated. The structure of apo-thrombin was obtained from the 4BAM structure by removing the ligand from the binding site and resolving with water molecules. Here, an equivalent number of water molecules at the surface of the solvation droplet were also removed in order to maintain an identical number of water molecules in the holo and apo systems. The same procedure was applied to get the same number of water molecules in the other holo systems. The systems with ligands free in water also had the number of water molecules as the plain water sphere.

### MD Simulations

MD simulations were performed with the Q software package (v5.10),^35^ applying the OPLS-AA/M force field.^36^ Interaction parameters to describe the three ligands were generated with Schrödinger’s ffld_server.^32^ The thrombin complexes were solvated in a spherical water droplet with a diameter of 70 Å that covers the entire protein, and Cl^–^ counterions were added to neutralize the protein net charge. The TIP3P water model^37^ was used, and water molecules at the sphere boundary were subjected to radial and polarization restraints following the SCAAS model.^35,38^ Lennard-Jones interactions were truncated beyond 10 Å, and long-range electrostatic interactions beyond this cutoff were treated with the local reaction field multipole expansion method.^39^ All MD simulations employed a 1 fs time step and were carried out in quintuplet (without hirugen) or triplicate (with hirugen) at five different temperatures between 283 and 303 K. The simulation protocol included initial structural minimization for 10 ps followed by a gradual heating to 293 K for 150 ps, with subsequent release of (10.0 kcal mol^–1^ Å^–2^) harmonic restraints on solute heavy atoms for 350 ps, followed by 0.5 ns equilibration at 293 K. The MD production runs to collect energy averages then involved 10 ns of simulation time for each replica at each temperature, giving a total of 5 replicas × 10 ns × 5 temperatures = 250 ns (without hirugen) and 3 × 10 × 5 = 150 ns (with hirugen) simulation time to calculate the heat capacity of each system.

### Free-Energy Calculations

Simulations of the mutation from compound **4** to **2** (Figure 2), whereby the terminal methyl group in the ethylamine substituent is perturbated into a hydrogen atom, were run in both the forward and reverse (hydrogen to methyl) perturbation directions starting from the prepared crystal structure 4BAN. The same transformations were repeated with the ligands free in solution to complete the thermodynamic cycle. These simulations were carried out with a 50 Å diameter sphere centered on the central amide oxygen atom, and protein atoms outside of this sphere were tightly restrained and excluded from nonbonded interactions.^35^ Ionizable residues close (<3 Å) to the system boundary were then taken in their neutral form to compensate for insufficient dielectric screening,^35^ and each system thereby had a total charge of +2 (the inhibitor charge). Each FEP calculation utilized 51 evenly distributed discrete sampling windows, with a 100 ps simulation time per window. The calculations were repeated 10 times with different random initial velocities, adding up to a total of 102 ns simulation time per leg of the thermodynamic cycle, including the forward and reverse directions.

## Supporting Information Available

A detailed description of free-energy perturbation calculations of the binding free-energy difference between ligands **2** and **4** as a function of temperature, with results summarized in Figure S1 (PDF)
