# Supplementary material for: Computational Analysis of Heat Capacity Effects in Protein–Ligand Binding
Source: J Chem Theory Comput. 2024 Jun 13;20(13):5708–16. doi: 10.1021/acs.jctc.4c00525 (PMC11238534; doi:10.1021/acs.jctc.4c00525)
Supplement: Supplementary file 1 — ct4c00525_si_001.pdf [file ct4c00525_si_001.pdf]

**Supporting Information**

# Computational Analysis of Heat Capacity Effects in Protein-Ligand Binding

Lucien Koenekoop and Johan Åqvist*

*Department of Cell & Molecular Biology, Uppsala University, Biomedical Center, SE-751 24 Uppsala, Sweden*

*Corresponding author:
aqvist@xray.bmc.uu.se

**Free energy perturbation calculations.** Since the three inhibitors have slightly different binding free energies and enthalpies, as measured by stopped-flow and SPR experiments,[5] it is also interesting to examine the performance of FEP calculations in this case. To this end, we calculated the binding free energy difference ($\Delta\Delta G_{bind}$) between compounds **2** and **4** that only differ by one methyl group. Here, ligand **4** is the tightest binding inhibitor and the measured $\Delta\Delta G_{bind} = -0.39$ kcal/mol with respect to **2**, from the SPR biosensor analysis at 25°C.[5] Moreover, the difference in binding enthalpy measured by ITC experiments ranges from $\Delta\Delta H_{bind} = -0.8$ to $\Delta\Delta H_{bind} = -0.4$ kcal/mol as the temperature is raised from 20°C to 35°C. We thus calculated the binding free energy difference between **2** and **4** by standard MD/FEP single topology calculations and repeated these simulations at five different temperatures (15, 20, 35, 30 and 35°C). At each temperature 10 replicate simulations were carried out to assess the convergence errors of the free energy estimates. In these MD/FEP simulations we used a smaller spherical droplet (diameter 50 Å) centered on the inhibitors, so that hirugen lies outside this sphere and does not affect the calculations.

The results from these MD/FEP simulations are shown in **Figure S1** as function of temperature. The calculated values of $\Delta\Delta G_{bind}$ are typically around –0.3 kcal/mol with relatively small standard errors of the mean (s.e.m.) of 0.3–0.4 kcal/mol at all temperatures. Hence, the overall value of $\Delta\Delta G_{bind}$ is basically reproduced by the calculations and for most purposes one would be happy with predictions that are within a few tenths of a kcal/mol of the experimental value. However, even with such rather precise free energy estimates, the temperature dependence of $\Delta\Delta G_{bind}$ is only qualitatively captured, at best, and predicts a slight decrease of the binding free energy difference with increasing temperature (**Figure S1**). This rather crude linear regression predicts a $\Delta\Delta H_{bind}$ value of about –2.3 kcal/mol, which clearly overestimates the experimental value.[5] Moreover, it is obvious that if there were a binding heat capacity difference between the two compounds, reflected by a curvature in the $\Delta\Delta G_{bind}$ vs. $T$ plot, this would be more or less impossible to estimate with any accuracy from these FEP calculations. Hence, it is tempting to conclude that $\Delta C_p$ differences between different ligands binding to a protein will be hopeless to calculate from the temperature dependence of free energies. On the other hand, in favourable cases it may be possible to reliably estimate $\Delta\Delta H_{bind}$ and $\Delta\Delta S_{bind}$ from computational van't Hoff plots.[16-18]

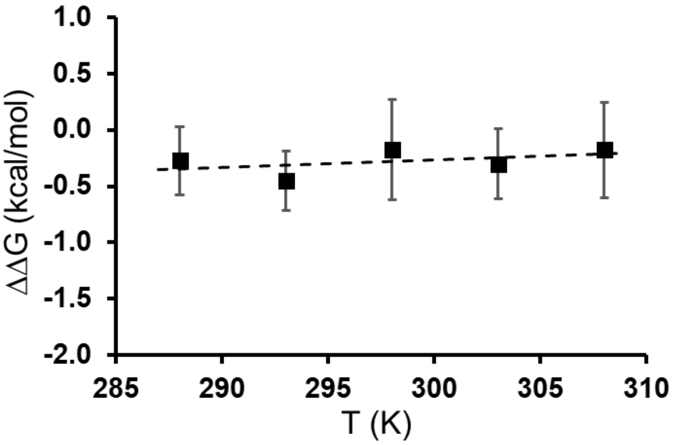


**Figure S1.** Relative binding free energies resulting from free energy perturbation calculations of transforming inhibitor **2** to **4**. Error bars denote ± 1 s.e.m. from 10 replicate simulations in both the forward and reverse perturbation directions. The predicted value of $\Delta\Delta G_{bind}$ at 25°C from linear regression is –0.3 kcal/mol and the corresponding experimental value is –0.4 kcal/mol.[5]

Here, it may be useful to again point out that for chemical reactions in enzymes and solution the analogous computational Arrhenius plots of activation free energy vs. temperature have proven to be extraordinarily accurate.[19-23] In these cases the free energy difference between the reactant and transition states is also calculated with FEP approach, where the reaction potential energy is described by the computationally efficient empirical valence bond method.[26] As noted above, what makes the FEP calculations considerably more accurate for chemical reaction steps than ligand binding is that no atoms are then created or annihilated during the transformation process. The only changes to the system in that case is that a few bonds are changed together with the charge distribution of the reacting fragments. Hence, the actual perturbations to the system are much smaller than when dealing with creation/annihilation of entire chemical groups.
